# Bryoherms from the lower Sarmatian (upper Serravallian, Middle Miocene) of the Central Paratethys

**DOI:** 10.1007/s10347-023-00661-y

**Published:** 2023-02-28

**Authors:** Werner E. Piller, Mathias Harzhauser

**Affiliations:** 1 Institut für Erdwissenschaften, NAWI Graz Geocenter, Universität Graz, Heinrichstraße 26, 8010 Graz, Austria; 2 Geologisch-Paläontologische Abteilung, Naturhistorisches Museum Wien, Burgring 7, 1014 Vienna, Austria

**Keywords:** Carbonate buildups, Bryozoans, Serpulids, Algae, Thrombolites

## Abstract

Bryozoan–serpulid–algal–thrombolite bioherms of up to 50 cm size are described from the Sarmatian (upper Middle Miocene) of the Central Paratethys. They occur on top of lower Sarmatian carbonate sediments of high-energy conditions and the individual bioherms settle on crests of ripples. The buildups are overlain and partly truncated by cross-bedded oolites of late Sarmatian age. Buildup growth starts with a *Cryptosula/Hydroides* (bryozoan/serpulid) pioneer community, followed by nodular *Schizoporella* (bryozoan) colonies overgrown by coralline algae/microbial mats and a thrombolite with calcareous algal filaments. All these constituents form a framestone fabric which is overall dominated by bryozoans labeling them as bryoherms. Inside the bioherms ecological successions of higher frequencies occur which are interpreted to reflect short-time environmental fluctuations such as nutrient availability, oxygenation (possible anoxia), salinity (possible brackish water), temperature and water level. The internal succession in individual bioherms is related to long-term environmental changes including general shallowing, increasing nutrient supply and decreasing water circulation and oxygenation. The described bioherms are most similar to modern bryostromatolites of the Coorong lagoon in S Australia and also similar to structures in the Netherlands. The widespread occurrence of bryoherms/bryostromatolites in the Central Paratethys suggests a phase of considerable eutrophication during the early Sarmatian.

## Introduction

Cenozoic carbonate buildups are dominated by corals which are capable to produce a variety of reefs of different sizes and shapes also known as coralgal reefs. Beside these classic reefs, non-coral bioconstructions also occur which may be dominated by different organisms, e.g., cyanobacteria, serpulid polychaetes, calcareous algae, foraminifera, bryozoans, vermetid gastropods, oysters (e.g., Wood 1999; Stanley 2001). These ‘atypical’ Cenozoic and modern reefs frequently grow in extreme environments as, for example, the well-known stromatolite reefs described from the Bahamas (e.g., Dill et al. 1986; Reid et al. 1995, 2011; Feldmann and McKenzie 1998).

Nevertheless, bryozoa are considered to be important carbonate producers in the Cenozoic (James 1997; Perrin 2002) representing for example main constituents of the bryomol facies (e.g., Nelson et al. 1988; Taylor and Allison 1998; Ernst 2020). Their reputation as frame-building organisms, however, is mostly based on their Paleozoic occurrences where some bioherms are entirely made up of bryozoans and, thus, called bryoherms (e.g., Cuffey 1977, 2006; Ernst 2020). Cenozoic and modern bryozoan reefs are reported mostly in nutrient rich, cool-water settings. Frequently cited are those from the Great Australian Bight where they form huge mounds up to 65 m thickness or even 200 m (Eocene mounds, Sharples et al. 2014) in water depths between 100 and 400 m (80–200 m, sea-level corrected) (Pleistocene, James et al. 2004, Quaternary, James et al. 2000). These bryozoan mounds, however, are composed widely of loose sediment with a high bryozoan share but form no framework/boundstone (e.g., Feary and James 1995; James et al. 2000, 2004; Sharples et al. 2014). Modern bryozoan reefs are more recently described from shallow water, c. 4–9 m from the south-eastern tip of Australia reaching a thickness of up to 1.5 m (Dutka et al. 2022). Modern reef pinnacles in which bryozoans dominate over corals are described from depths between the 2 and 25 m from the Abrolhos Shelf off Brazil in the South Atlantic (Bastos et al. 2018).

Complex modern bioconstructions dominated by bryozoan colonies are described from somewhat extreme environments: briefly described are those from Joulters Cays (Great Bahama Bank) where they occur in a shallow water tidal channel exposed to high water energy in an area of oolite production. Such environmental conditions are unfavorable for coral growth and forced the ecologically broader adapted bryozoans to flourish (Cuffey and Gebelein 1975; Cuffey et al. 1977, 1979; Cuffey and Fonda 1979). Bioconstructions with dominant bryozoans were also described from the southern Australian Coorong lagoon. They have been named either as bryozoan–serpulid buildups (Bone and Wass 1990) or subsequently as bryostromatolites (Palinska et al. 1999; Scholz 2000; Scholz et al. 2000, 2005). These also occur in an extreme environment concerning values and changes in water depth, temperature and salinity.

Modern microbialites with bryozoan and serpulids were described from brackish inland lakes from the Netherlands. These structures were already known in the seventeenth century as “growing stones” and described in detail by Bijma and Boekschoten (1985) as bryozoan/stromatolite reefs. These have more recently been re-studied with modern documentation and analytical methods by Harrison et al. (2021) denominated as bryostromatolites (“bryoliths”).

From the Ligurian Sea in Italy (Mediterranean) bryozoan bioconstructions were described from water depth ranging between 0.3 and 8 m composed predominantly by the bryozoan species *Schizoporella errata* (Cocito et al. 2000). Cocito (2004) gives an overview on bryozoan bioconstructions globally both on the bryozoan frame builders but also associated biota. In addition, Mediterranean microbialites were described from coastal lagoons/ponds from Sardinia and France (Saint Martin and Saint Martin 2015a, b).

Concerning the Cenozoic fossil record, Messinian (Late Miocene) reefs in the Mediterranean are frequently reported, in particular from Spain (Martín et al. 1997; Braga et al. 2006, 2009) and from Italy (Moissette et al. 2002; Bosellini et al. 2001; Braga et al. 2009). These are mostly dominated by corals but also reported as bivalve–bryozoan–serpulid reefs (Braga et al. 2009). Additionally widespread are microbial reefs mostly related to coral reefs with a variable share of corals, serpulids and bryozoans (Saint Martin et al. 1996; Saint Martin 2001; Moissette et al. 2002; Vescogni et al. 2022).

Neogene distinctly bryozoan-dominated buildups lacking corals are hitherto only cited to form ‘reefs’ or ‘biostromes’ or ‘biolithites’ in the Sarmatian of the Central Paratethys (Middle Miocene) and from the Middle to Upper Miocene of the Eastern Paratethys (e.g., Andrus(s)ov(w) 1899–1909; Andrusov 1936; Małecki 1980; Pikija et al. 1989; Pisera 1996; Bucur et al. 1992; Friebe 1994a, b; Harzhauser and Piller 2004a, b; Piller and Harzhauser 2005; Cornée et al. 2009; Saint Martin and Saint Martin 2015b). Herein, we describe bryozoan-dominated bioconstructions from the Vienna Basin (Central Paratethys) providing a detailed description of the internal composition and growth succession and discuss the paleoenvironment in which these bioconstructions may have formed in comparison with possible modern counterparts.

## Historical background of Sarmatian bryozoan-rich reefs in the Paratethys

**Fig. 1:**
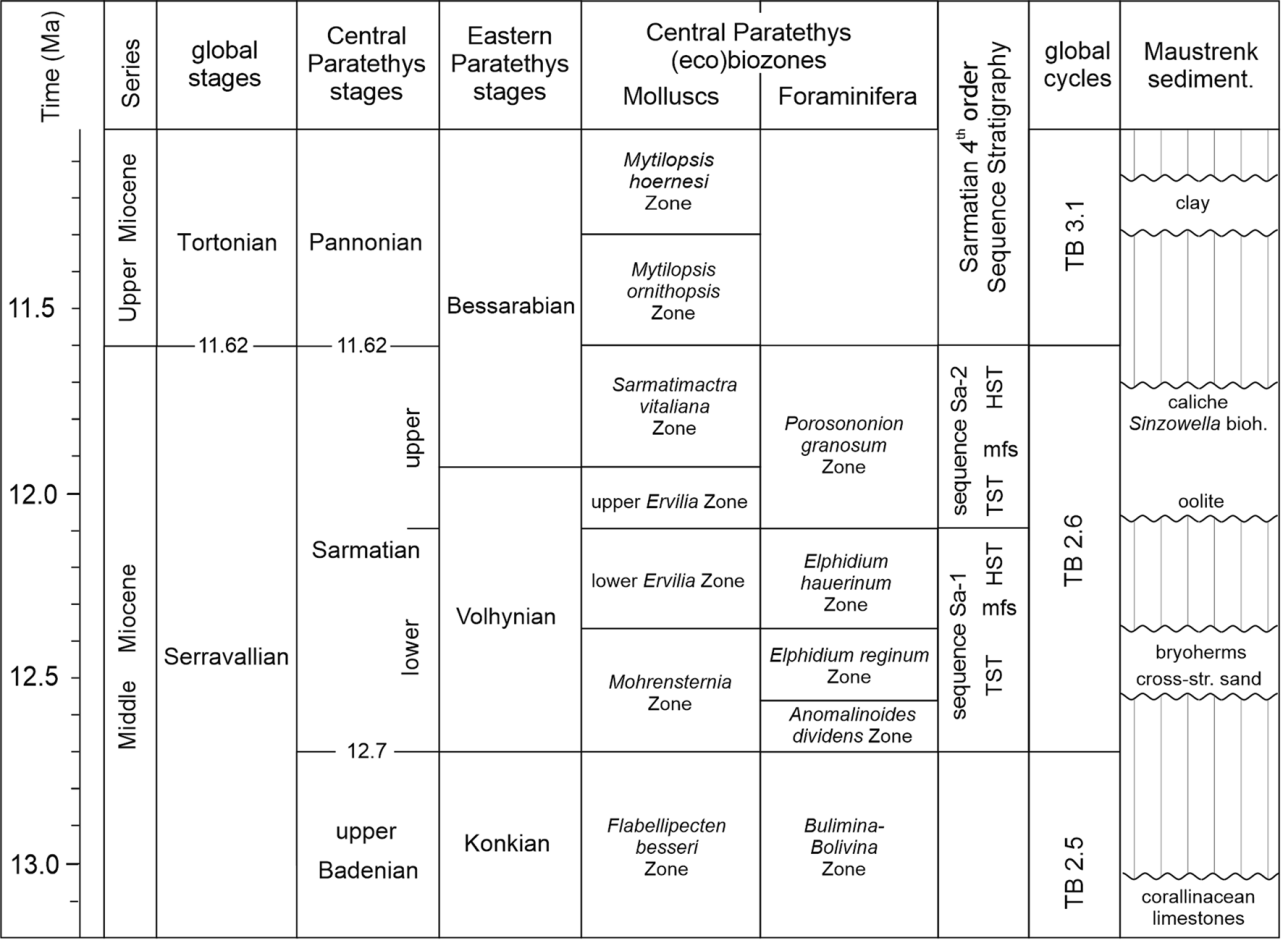
Middle/Upper Miocene chronostratigraphy, biostratigraphy and sequence stratigraphy of the Mediterranean and Paratethys (after Harzhauser and Piller 2004a, b; Piller and Harzhauser 2005; Strauss et al. 2006; Piller et al. 2007; Siedl et al. 2020; Kranner et al. 2021a; global sequences after Haq et al. 1988 and Hardenbol et al. 1998). The right column represents the sediment sequence at the studied location reflecting the various gaps between the lithologic units

The first known report on bryozoan reefs in the Sarmatian came from the Eastern Paratethys (nowadays Poland, Ukraine, Moldova, Romania, Bulgaria, Russia) communicated by Barbot de Marny (1866, p. 340) who even used the expression bryozoan atolls and atolls of *Eschara lapidosa* (1867, p. 174). Olszewski (1875) used the descriptive name “Kalkstein mit *Serpula gregalis* Eichw.” but was in favor of “Tarnopoler Brackwasserkalkstein” (brackish water limestone of Tarnopol). Hilber (1882) substituted the term Serpula limestone (“Serpulenkalk” of Pusch 1836) by the name Pleuropora limestone after the bryozoan species *Pleuropora lapidosa* Pallas (actual name *Tamanicella lapidosa* after revision by Viskova and Koromyslova 2012). Teisseyre (1884) studied the hill range Medobory in Podolia (Ukraine) and described Sarmatian Pleuropora limestones of 20–80 m thickness which he also interpreted as bryozoan reef. The lateral extension reaches up to 4 km and he speculated that these reefs were already subaquatic ‘islands’ during growth which were surrounded by loose Sarmatian sands. Andrus[s]ov[w] (1897, 1899, 1909, 1936—to mention only some of his publications) published extensively on the Sarmatian of the Eastern Paratethys and in particular on bryozoan reefs (1909–1912). A first modern overview on Paratethyan reefs including Sarmatian ones was published by Pisera (1996). The major differences between Sarmatian bioconstructions between the Eastern and Central Paratethys is their spatial distribution and size as well as their stratigraphic range (see below). The individual reefs in the Central Paratethys are small (decimeter—rarely larger than 1–2 m) and are spatially very restricted (meter scale) and occur only in the Sarmatian because the Pannonian is already represented by the brackish to freshwater Lake Pannon (Fig. 1). In the Eastern Paratethys, the reefs can reach tens of meters in thickness, show laterally a wide distribution of tens of kilometers and occur from the Volhynian to the Khersonian.

In the Eastern Paratethys the best studied reefs occur in the Ukrainian Medobory Hills (Saint Martin and Pestrea 1999; Jasionowski 2006; Taylor et al. 2006; Studencka and Jasionowski 2011; Jasionowski et al. 2006; Górka et al. 2012) and on the Kertsch and Taman peninsulas (Abich 1865; Andrussow 1909; Goncharova and Rostovtseva 2009). The reefs in the Medobory Hills are called ‘serpulid–microbialite reefs’ but microbialites dominate by far and serpulid tubes and bryozoa are subordinate (e.g., Jasionowski 2006; Górka et al. 2012). In her studies on trochid gastropods, Sladkovskaya (2017) covered a large area from the Carpathian Foredeep to the Caspian Sea–Lake Aral area and also mentioning reef buildups from many study sites.

In the Central Paratethys, descriptions of Sarmatian reefs are relatively rare compared to the Eastern Paratethys. Pikija et al. (1989) described three biolithite facies (bryozoan–corallinacean, corallinacean, and bryozoan biohermal bodies) from Krašić (Croatia), however, a differentiation between lower and upper Sarmatian was not possible. Bucur et al. (1992), as an exception, described Sarmatian reefs both from the Carpathian Foredeep and the Pannonian Basin System from Romania; the Central Paratethyan reefs were studied in the Şimleu Basin which have been re-studied later on by Daoud et al. (2006). The most detailed description and environmental interpretation of such structures from the Styrian Basin was provided by Friebe (1994a, b), the nature of the outcrop and complexity of the geological structures, however, obscured important features (Harzhauser and Piller 2004a). Harzhauser and Piller (2004a, b) re-studied these outcrops and additional ones in the Vienna Basin clarifying stratigraphy and offering new details. Cornée et al. (2009) described bioherms from the Pannonian Basin of Hungary with rare occurrences from the lower Sarmatian and frequent ones from upper Sarmatian. Hyzny et al. (2012) briefly mentioned bryozoan–serpulid limestones from the eastern margin of the Vienna Basin in Slovakia.

Sarmatian bryozoans have variously been described by, e.g., Małecki (1980), Taylor et al. (2006), Zágoršek and Fordinál (2006), Zágoršek (2007), Zágoršek et al. (2008), and Hara and Jasionowski (2012). These contributions focus on taxonomy and early ontogenetic development but did not describe multi-species colonies and bryoherm formation.

## Paleoenvironmental and paleogeographic setting and stratigraphic frame

The Paratethys was born when the Western Tethys was subdivided into a southern and a northern part due to the uplift of the alpine chains at the Eocene/Oligocene boundary (Rögl and Steininger 1984; Rögl 1996, 1998, 1999). The southern part evolved into the Mediterranean Sea, the northern part became the Paratethys. Due to the geodynamic evolution of the area and to global sea-level fluctuations the connections between the Paratethys and the Mediterranean but also to the North Sea and the Indian Ocean frequently changed (Rögl 1998; Popov et al. 2004). This led to a partly independent development of both parts which is expressed in the establishment of different chronostratigraphic/geochronologic scales (Fig. 1). However, even between the Western/Central and Eastern Paratethys, a differing chronostratigraphic/geochronologic subdivision was introduced (Fig. 1).

The only interval when the Paratethys represented a palaeobiogeographically uniform entity was in the late Serravallian based on the occurrence of identical or similar biota distributed from the Vienna Basin in the west to the Caspian Basin in the east (e.g., Papp et al. 1974; Rögl 1998; Harzhauser and Piller 2007). This biota (also called “Cerithienschichten”) was the reason for Suess (1866) and Barbot de Marny (1866) to introduce the name Sarmatian for this interval covering the upper Middle Miocene in the Central Paratethys. A major change occurred with the onset of the Late Miocene, when the marine environment was terminated in the Central Paratethys and Lake Pannon became established. In the Eastern Paratethys, however, the marine environment continued. This led to different sedimentary successions in the Central and Eastern Paratethys and, as a consequence, the regional chronostratigraphic stages Volhynian, Bessarabian and Khersonian were established for the Eastern Paratethys whereupon the Volhynian approximates the lower Sarmatian of the Central Paratethys and the lower Bessarabian covers the upper Sarmatian (Fig. 1); the upper Bessarabian belongs already to the Late Miocene as does the Khersonian (e.g., Papp et al. 1974; Paramonova 1974; Harzhauser and Piller 2004b, 2007; Piller and Harzhauser 2005). The Sarmatian stage was clearly defined for the Central Paratethys, nevertheless, for the Volhynian, Bessarabian and Khersonian the name Sarmatian s.l. is still in use (e.g., Gradstein et al. 2020) but has to be avoided to prevent misunderstandings. In addition, geotectonic processes strongly influenced the paleogeography of the Paratethys producing different distributional patterns through time. In the Sarmatian, the Carpathian Foredeep was, in particular, affected. During the Badenian, the Carpathian Foredeep was connected with the Vienna Basin and did belong to the Central Paratethys. In the Sarmatian, it became disconnected and, consequently, necessitates the classification of the sediment successions of the Carpathian Foredeep and the connected Dacian Basin with the regional stratigraphic stages Volhynian, Bessarabian and Khersonian what also clearly assigns this area (including parts of Poland, Ukraine, Moldova, Romania and Bulgaria) to the Eastern Paratethys (e.g., Studencka and Jasionowski 2011; Sladkovskaya 2017; Koleva-Rekalova and Darakchieva 2020). The Central Paratethys consisted in the Sarmatian only of the Vienna Basin, the Pannonian Basin system (including the Styrian Basin) and the Transylvanian Basin.

In the Central Paratethys, the Sarmatian was and still is generally interpreted as transitional from the marine Badenian sea towards the temperate-freshwater environments of Lake Pannon (Papp 1954, 1956). This interpretation was mainly based on the absence of stenohaline biota, such as radiolaria, planktic foraminifera, corals and echinoderms (comp. Steininger and Wessely 2000), which disappeared at the Badenian/Sarmatian boundary, what is still common scientific knowledge (e.g., Rögl and Steininger 1984; Rögl 1996; Kováč et al. 1999). Contrary to the brackish water interpretation, already Belokrys (1967) and later on Pisera (1995, 1996) suggested a high saturation of Ca-carbonate and high alkalinity for the Sarmatian and proposed locally even hypersaline conditions. Harzhauser and Piller (2004a, b) and Piller and Harzhauser (2005) performed an integrated study on the Sarmatian of the western Central Paratethys relying mainly on the Vienna and Styrian basins and the North Alpine Foreland Basin and were able to demonstrate a highly differentiated and complex development during this time interval (see below). In the Vienna Basin, the Sarmatian is sequence stratigrapically bounded by a 3rd-order sequence boundary at its base and top (Harzhauser and Piller 2004b; Strauss et al. 2006; Piller et al. 2007) which can be correlated with the TB 2.6 global cycle of Haq et al. (1988) and Hardenbol et al. (1998) with the base representing lowstand Ser3 and at the top lowstand Ser4/Tor1 (Fig. 1). Internally, a strictly twofold subdivision occurs reflecting two 4th-order cycles—the lower Sarmatian cycle Sa1 and the upper Sarmatian Sa2 (Fig. 1). The cycle Sa1 is mainly a siliciclastic cycle, Sa2 is a mixed siliciclastic/carbonate cycle. Both 4th-order cycles are separated by an extensive erosional phase during which lower Sarmatian carbonates were reworked and later on deposited in upper Sarmatian sediments. This erosional phase is also reflected in some places by paleokarst features and formation of pedogenic carbonate with caliche and *Microcodium* (Harzhauser and Piller 2004a). In coastal surface outcrops, the lower Sarmatian is characterized by gravely/pebbly sediments and siliciclastic tidal flat deposits with a peculiar gastropod fauna (e.g., *Mohrensternia* div. sp.). In more basinal positions, marine diatomites and marls occur (Schütz et al. 2007). Carbonates occur as nearly monospecific serpulid-beds made up by *Hydroides* in basin central locations and as serpulid–bryozoan–microbial bioconstructions (see below) in locations close to the paleocoast (Harzhauser and Piller 2004a). Above the erosional surface mentioned before, the upper Sarmatian starts with predominantly carbonate sediments made by up to 30 m thick oolites of Persian Gulf type, by up to 10 m thick molluscan shell coquinas (Harzhauser and Piller 2009) and typical foraminiferan–algal-bioconstructions with characteristic nubeculariid foraminifers (*Sinzowella*) (Fig. 1). Also, very typical for the upper Sarmatian are beds with mass occurrences of the larger foraminifer *Spirolina austriaca*, which serve as excellent correlation horizons within the basin (e.g., Piller and Harzhauser 2005). Many biota and sedimentary features (diatomites, larger foraminifers, molluscs) clearly prove a fully marine environment for most or at least parts of the Sarmatian. Only locally, in coastal areas, brackish water environments have occurred; however, in many other locations, particularly in the upper Sarmatian, hypersaline conditions prevailed (Latal et al. 2004; Piller and Harzhauser 2005). In coastal positions, lower Sarmatian serpulid–bryozoan–microbial carbonates directly underlie upper Sarmatian carbonates—oolites as well as nubeculariid bioherms—with an often unclear boundary in between due to the erosional phase (Fig. 1). This immediate physical neighborhood produced some confusion and—in combination with bad outcrop situations—led to an intermingling of lower and upper Sarmatian carbonates (e.g., Nagy et al. 1993; Friebe 1994a).

## Methods

The studied succession was logged in detail in the field, documented and several samples were taken out of each bed. Samples of limestone and cemented sandstone beds were thin-sectioned. Buildups were taken as a whole and sawed into c. 4–5 cm thick slabs and some were polished. The composition and succession within the slabs were graphically documented. Some of the slabs were also thin-sectioned to study the internal composition. This information was also combined with information in the graphically documented slabs. About 50 thin sections were prepared sized 5 × 5 cm and 10 × 6 cm. Thin-section studies were carried out with a Leica MZ16 und a Zeiss Axioplan2 microscope using the ImageAccess ver. 5 rel. 186 for measurements as well as with a digital microscope Keyence VHX-6000 with included software. Growth form of bryozoans follows Taylor and James (2013).

## Results

### Study area and general setting

**Fig. 2:**
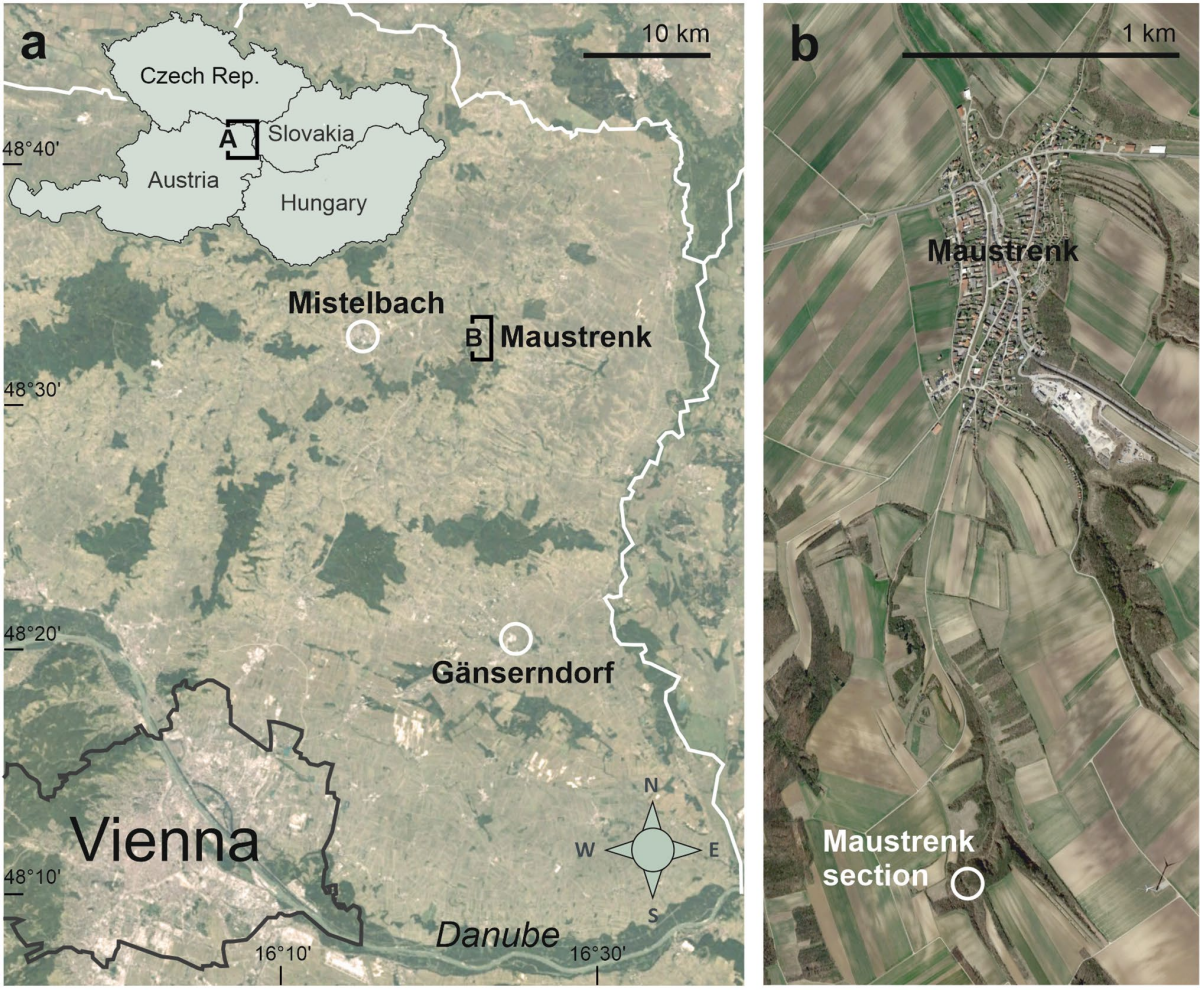
**a** Locality map showing Maustrenk (Lower Austria) in the northern Vienna Basin. **b** Position of the studied buildups south of Maustrenk (Maps generated with Google Earth Pro, Image © 2022 Maxar Technologies)

The study area is located close to the village of Maustrenk (Lower Austria, about 50 km NE of Vienna) at the termination of a short and narrow valley called “Steingräben” (016°42,06′E, 48°33,23′N) (Fig. 2).

**Fig. 3:**
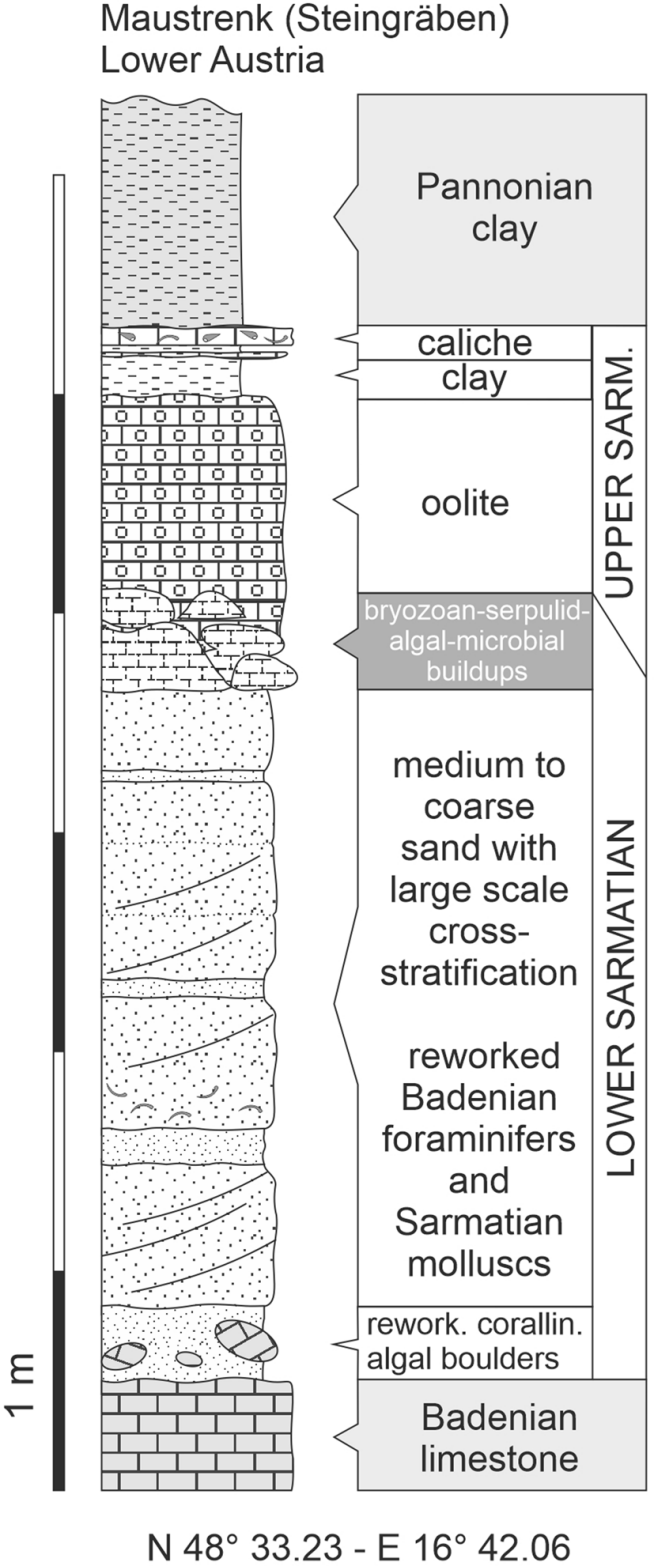
Lithologic succession and chronostratigraphic assignment of the outcrop at Maustrenk. Scale in meters

**Fig. 4:**
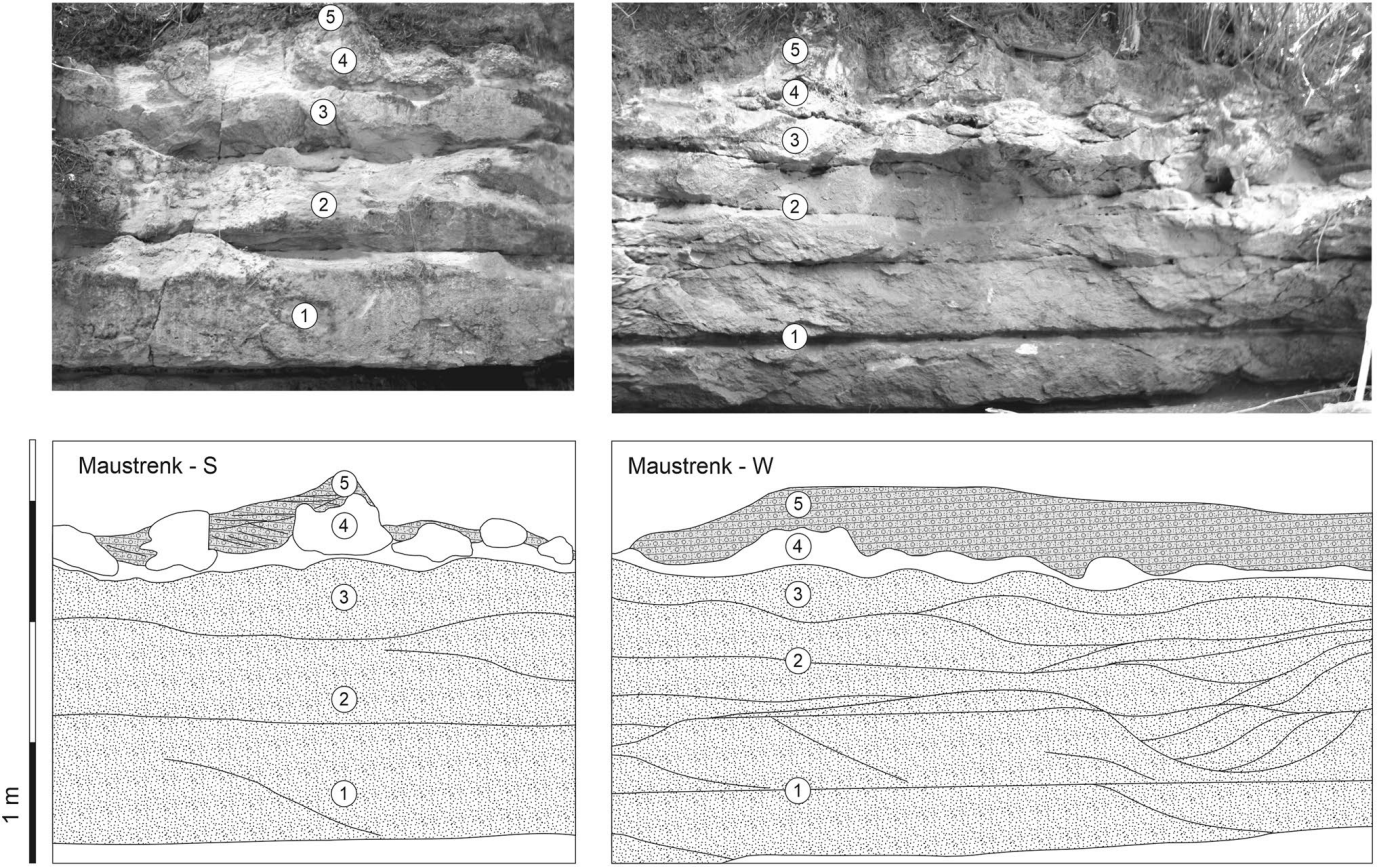
Outcrop wall at Maustrenk (Lower Austria). At the very base, the Badenian Leitha Limestone is truncated by a disconformity. Sarmatian sediments start with cross-bedded, channelized coarse-grained carbonate sands and detrital carbonate sand dunes (1, 2) which are topped by a bed with ripples (3). The ripples are settled by bryozoan-dominated buildups. After an erosional surface, space between buildups is filled by cross-bedded oolites (4) which also form the top bed (5)

**Fig. 5:**
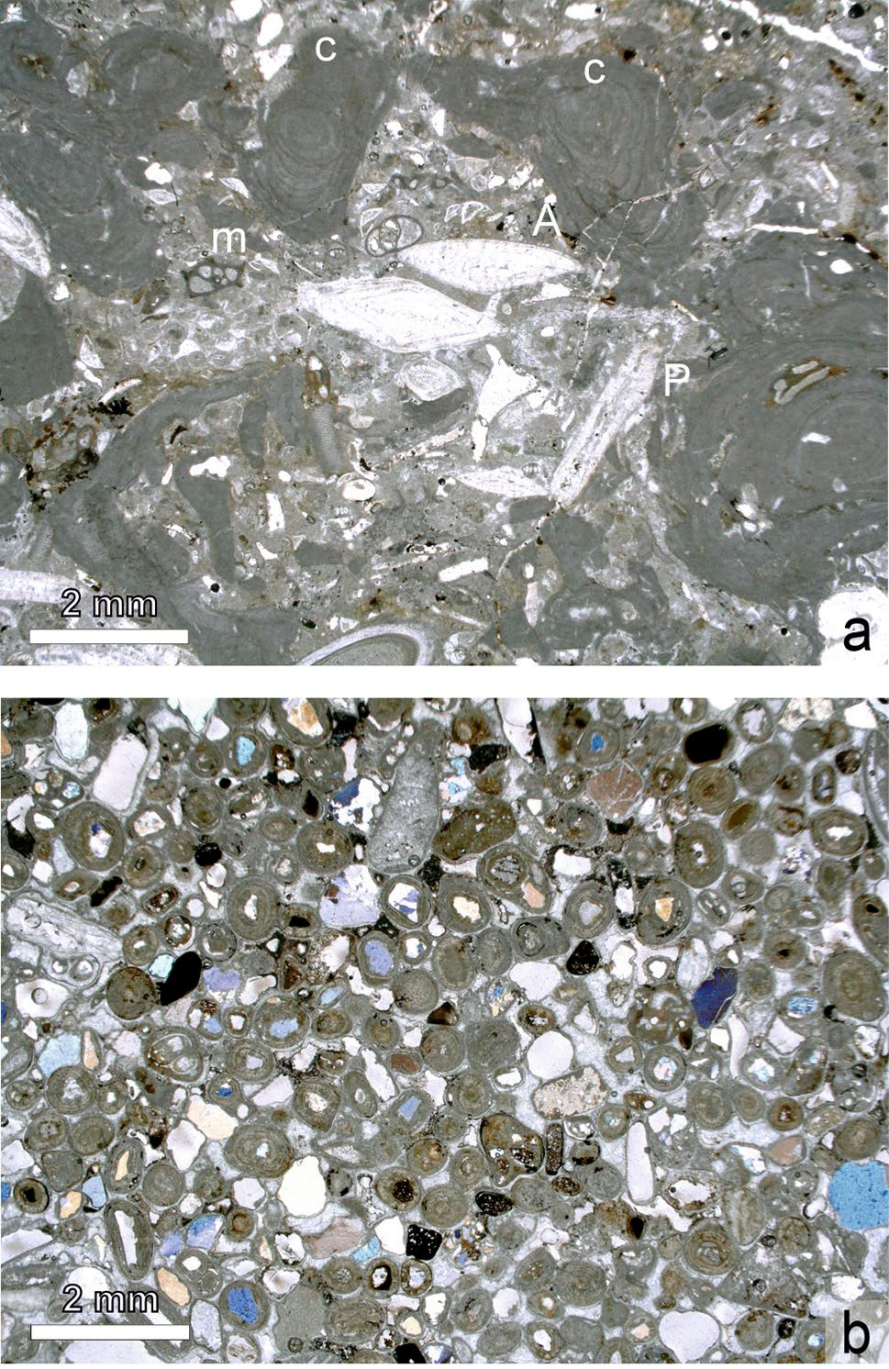
**a** Microfacies of the Badenian Leitha Limestone with larger foraminifers (*Amphistegina*, A, *Planostegina, P*), coralline algal branches (c) and miliolid foraminifers (m). (Thin section, sample MT1) **b** Microfacies of oolite grainstones overlying the bryozoan buildups. (Thin section, sample MT14-03)

The sedimentary sequence in which the buildups occur is about 6–7 m thick (Figs. 3, 4). It starts at the base with Middle Miocene (Badenian) Leitha Limestone made up predominantly by coralline algal fragments and larger foraminifers (*Amphistegina, Planostegina, Borelis*) (corallinacean–foraminiferal rudstone) (Fig. 5a).

Above a disconformity, visible by a weak relief, an about 3–4 m thick, well-bedded limestone sequence follows. The basal bed (below 1 in Fig. 4, right panel) is of about 30 cm thickness; however, thickness changes laterally due to the uneven surface of the underlying bed. It is a coarse calcareous sandstone of mainly well-rounded coralline algal grains with big blocks of up to 50 cm diameter of mostly well-rounded underlying Leitha Limestone with indistinct cross-bedding.

Upsection follows coarse, well-rounded, weakly cemented detrital carbonate sands to sandstones (below 2 in Fig. 4, right panel) arranged in large, cross-bedded sets with troughs of < 60 cm. These troughs are oriented SSW–NNE. These distinctly cross-bedded and channelized carbonate sandstone beds are overlain by beds with large ripples (height: 20 cm, amplitude: 90 cm) exhibiting roughly the same orientation as underlying troughs (3 in Fig. 4).

On top of many ripples, individual carbonate buildups occur (4 in Fig. 4). The space between the buildups is filled by cross-bedded, weakly cemented, quartz-rich oolite sand and the overlying bed covering the buildups is also a cross-bedded sand made up predominantly of multi-layered, radial oolites with quartz-nuclei (5 in Figs. 4, 5b). The oolites reach up a thickness of 1 m. The buildups and sediments forming their substratum are truncated and/or eroded prior to sedimentation of oolite sands.

The section above the oolites is rather poorly outcropping but is made up of fine siliciclastics with abundant cardiid bivalves in life position (*Plicatiforma latisulca*) intermingled with or grading into a few centimeters of caliche crusts. This sediment is overlain by a quartz- and lithoclast-rich oolithic sandstone to molluscan packstone–rudstone with very abundant spirorbids. Within these sandstones and limestones nubeculariid bioherms up to 50 cm in height with columnar growth form occur. The top of the section is represented by badly outcropping Pannonian (Late Miocene) clay (Fig. 3).

### Buildups

**Fig. 6:**
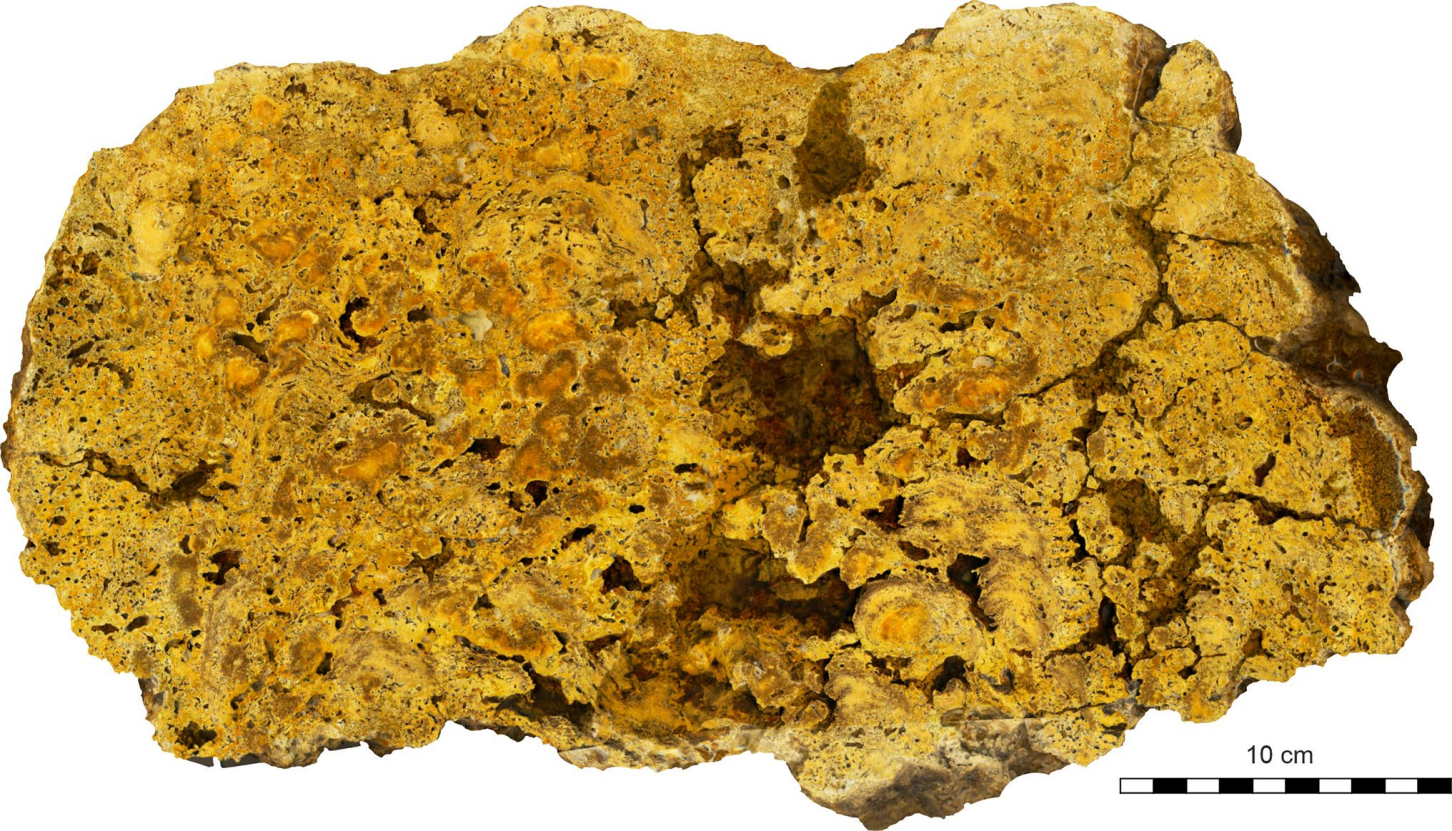
Cross section of an individual bryoherm (cut and polished slab)

The buildups are globular, lense-like or columnar in shape and reach more than 50 cm in height and width. Along the outcrop of approx. 20 m 8–9 individual buildups can be observed (Fig. 4). Vertical sections through individual buildups clearly show a boundstone with large open pore space and cavities (Fig. 6). Cavities are irregular in shape and up to several centimeters wide. The boundstone has a knobby to nodular appearance with individual knobs a few centimeters in height and diameter. The knobs frequently have a dense ‘core’ and a less dense periphery and exhibit a more or less distinct lamination. Between the knobs and the top of the buildups highly porous limestone occurs.

**Fig. 7:**
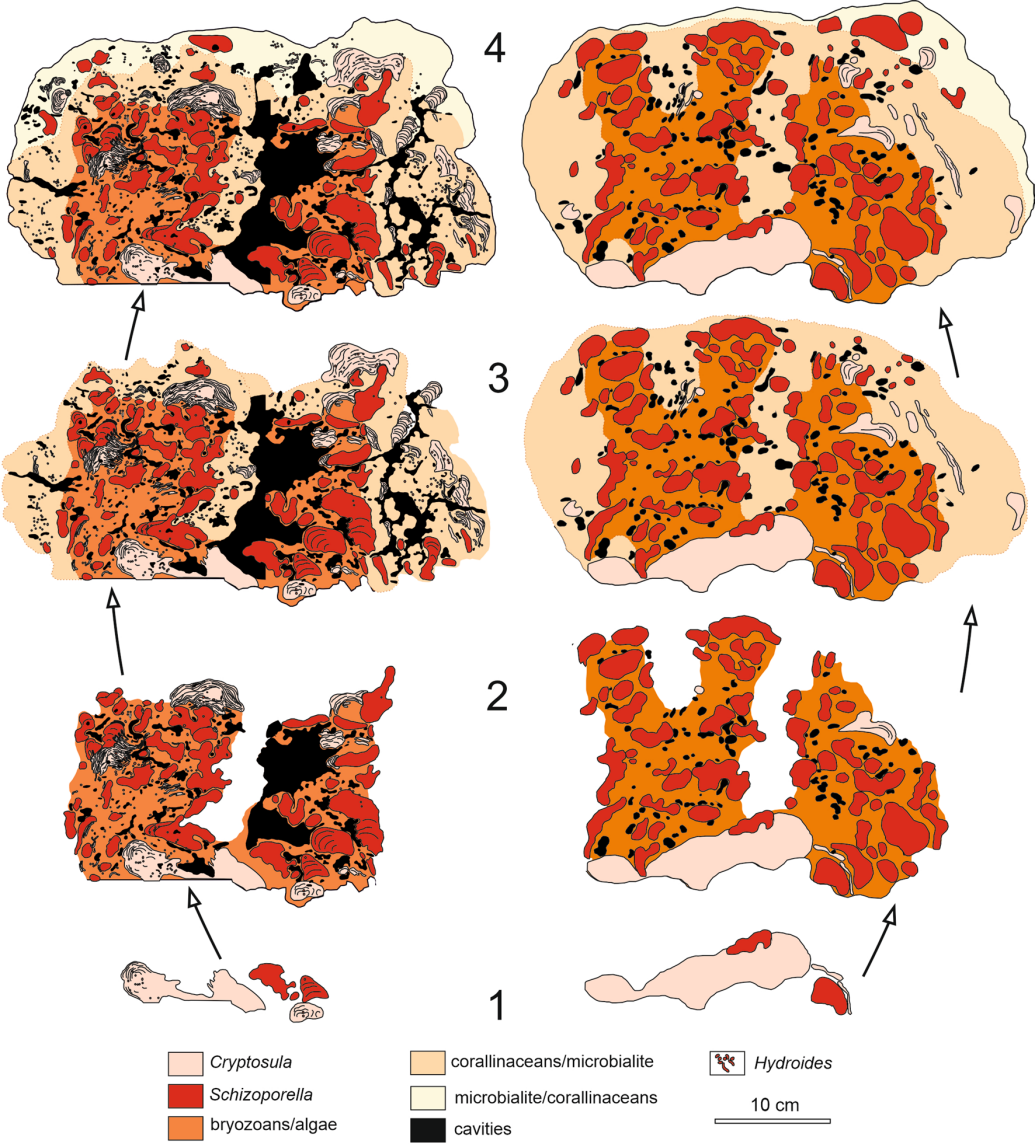
Drawing of coinciding internal construction, depicting ecological successions of two consecutive cross sections of the same bryozoan–algal–thrombolite buildup. The succession to the left represents the slab shown in Fig. 6. In some areas, differentiation between corallinacean and microbial carbonate is not possible from the surface; therefore, these are combined and only differentiated by the dominant group, respectively. Note that the structure initially consisted of two individual buildups (1, 2) which coalesce during a later growth stage (3, 4)

**Fig. 8:**
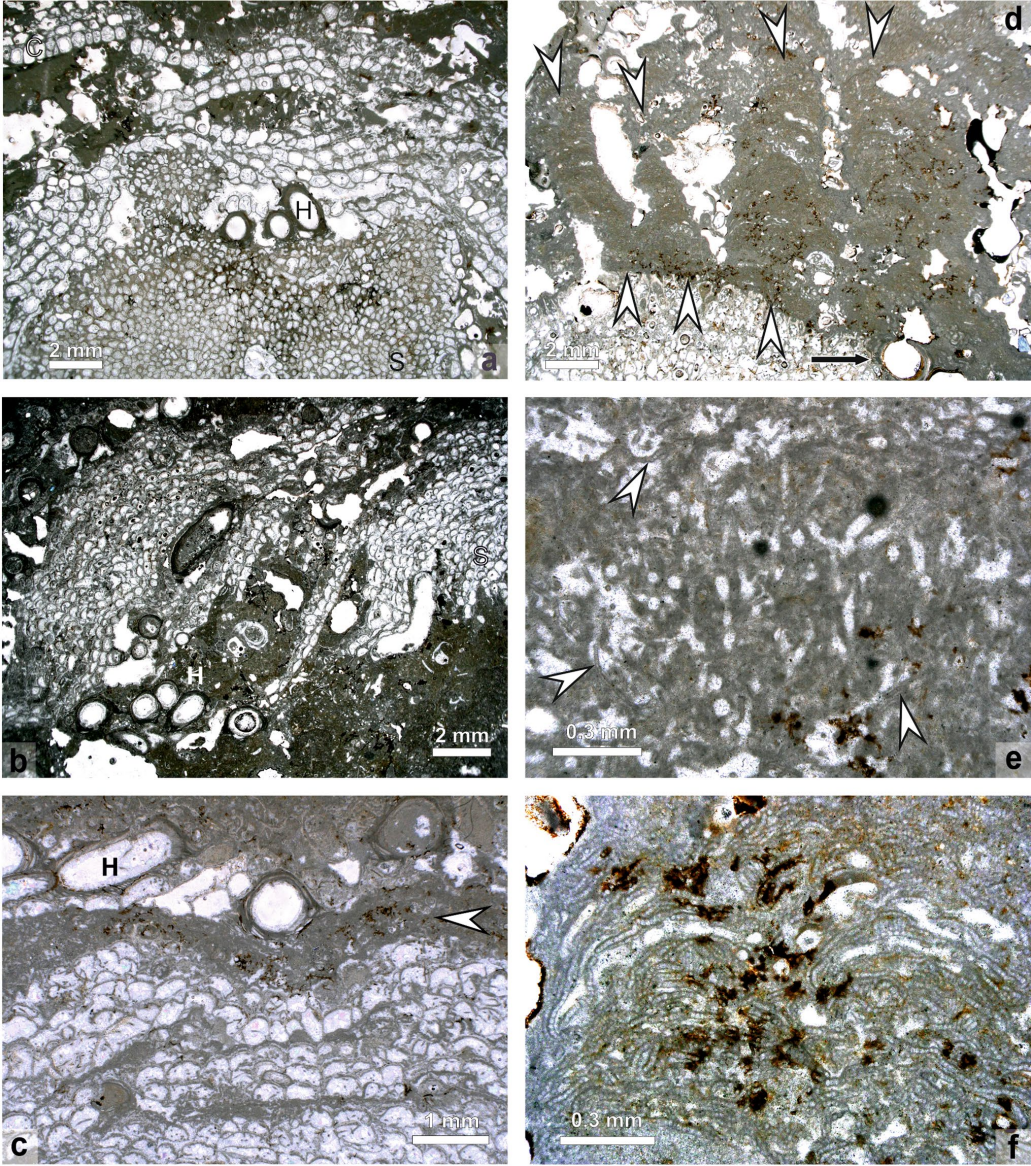
Microphotographs of internal structures of buildups. **a** *Schizoporella* (S) colony (base) overgrown by *Hydroides* (H) and crustose bryozoan *Cryptosula* (C) (thin section MT5) **b** Repeated succession of *Schizoporella* colonies (S) and coralline algae/thrombolites. Frequently, *Hydroides* tubes (H) are interbedded in *Schizoporella* colonies and coralline algae/thrombolite carbonate (thin section MT8c/02). **c** Bryozoan colony overgrown by coralline algae (arrowhead) and *Hydroides* tubes (H) (thin section MT) **d** *Schizoporella* colony overgrown by columnar coralline algae (arrowheads) giving the columns a stratified appearance (thin section MT7) **e** Calcified, branching filaments of eukaryotic (?) algae in micritic and clotted sediment of thrombolitic parts of the buildups; between these filamentous structures coralline algal filaments occur (arrowheads) (thin section MT 8b) **f** Detail of coralline algal filaments or sheets with dusty, clotted micrite in between (thin section MT7)

Finely ground slabs through the buildups allow a rough assignment of major biotic components (Figs. 6 and 7). The basal part is constructed by sheet-like cheilostome bryozoan colonies building up several centimeters of skeletal carbonate by forming repeated crusts (Fig. 7). Applying the taxonomy currently in use for these bryozoa they have to be placed into the genus *Cryptosula* (Fig. 8a) (e.g., Bobies 1957; Ghiurca and Stancu 1974; Vávra 1977; Pisera 1996). The serpulid *Hydroides* is frequently intergrown with *Cryptosula.* This *Cryptosula*-bindstone is overgrown by and provides substrate to globular to hemispherical colonies of the multi-layered bryozoan genus *Schizoporella*, which makes up the bulk of the buildup (Fig. 7), forming a rigid framestone. The individual *Schizoporella* colonies reach up to 5 cm and more and are also frequently inter- and overgrown by *Hydroides* (Fig. 7). In many places, this *Schizoporella-*framestone is over- and intergrown with coralline algae or cryptocrystalline calcite, which form abundant columnar protrusions up to several centimeters in thickness (Fig. 7). This *Schizoporella*/algal-framestone (bryozoan and algae cannot be separated macroscopically and are treated as one unit in Fig. 7) occurs in repeated successions and is covered by a microbial carbonate (Fig. 7). The overall growth form of the *Schizoporella*/algal-framestone is columnar (Fig. 7). The columns are smoothened later on by the terminating microbial carbonate (Figs. 6 and 7). Within the framestone rare gastropods (*Gibbuliculus* sp.) and bivalves (*Musculus sarmaticus*) occur.

In thin section, the macroscopical observations can be verified, but the succession is much more complex. The buildup starts with a *Cryptosula*-bindstone which contains also *Hydroides* tubes. On top of the encrusting-foliose *Cryptosula* layers the domal (knobbly and columnar) *Schizoporella* colonies follow, which are also frequently grown around or intergrown with *Hydroides* tubes (Fig. 8a–c). *Schizoporella* itself is overgrown either by filamentous structures (Fig. 8c, e) or by a succession of *Hydroides* and filaments, which again may be followed by *Cryptosula* (Fig. 8c). The filamentous structures on the *Schizoporella* colonies are indistinctly cellular and can be attributed to coralline algae (*Lithoporella?*) (Fig. 5f). These algal thalli are either single filaments (filament diameter: 12–23 µm, cell length: 15–25 µm) or single-layered sheets, but they mostly do not form a vertical continuous and densely packed sequence but occur mostly spaced from each other, surrounded by a first cement generation and a fuzzy, indistinctly defined, dusty microcrystalline calcite (Fig. 8f).

**Fig. 9:**
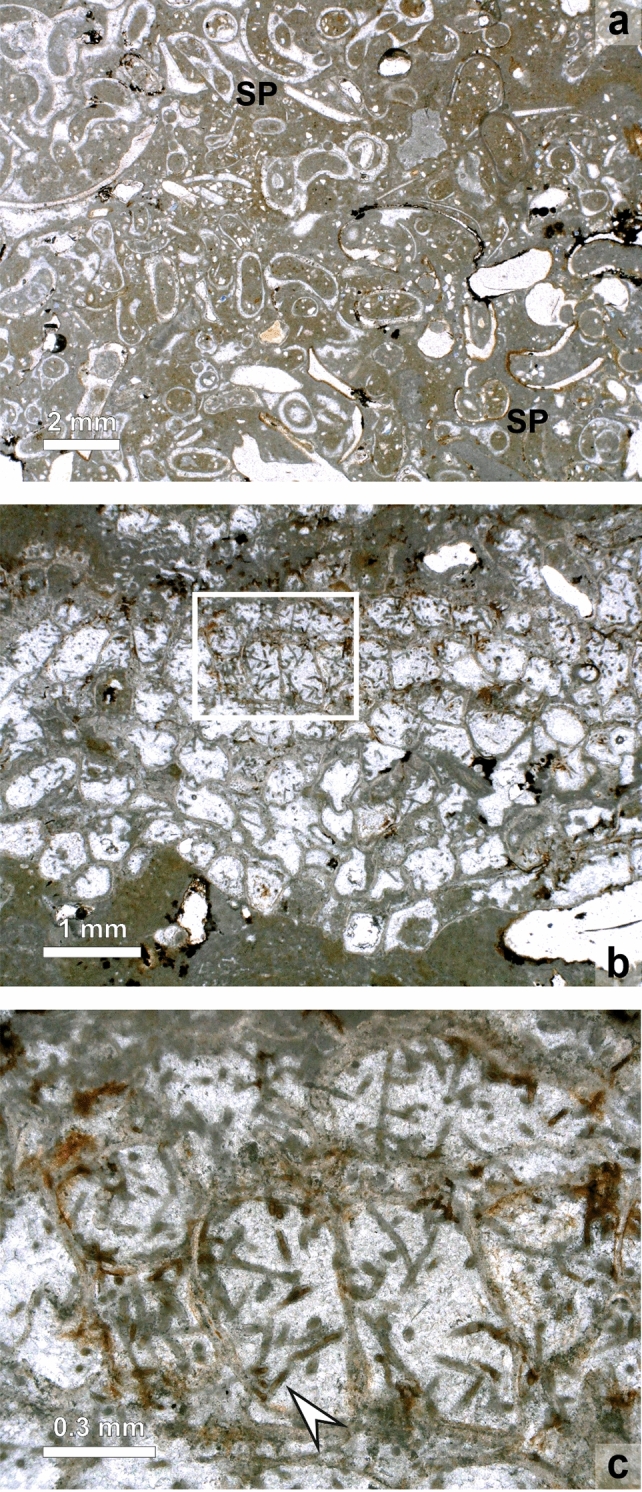
**a** Microfacies of *Spirorbis* (SP) dominated biomicrite building the top of the bryozoan buildups (thin section MT14-3). **b** Microphotograph of zooecia of bryozoans with internal branching filaments** c** Enlargement of the filaments of Fig. 9**b** (white rectangle); the filaments are overgrown by a first cement generation (arrowhead) (thin section MT8-b1)

The coralline algal bindstones become thicker close to the outer surface of the buildups and the overall growth form is columnar with protrusions up to several centimeters in height (Fig. 8d). Within this bindstone bifurcating filaments without any cellular subdivisions occur (see also below) (Fig. 8e). Outwards of these filaments again *Cryptosula* and *Hydroides* may dominate. Finally, the framestone is covered by a cap dominated by bifurcating filaments (two size categories in diameter: 15–20 µm; 40–60 µm) (Fig. 8e) in a clotted, micritic matrix which is best described as thrombolite (sensu Riding 2000). In some places, spirorbid serpulids may be abundant in the marginal (outer) part of the buildups (Fig. 9a). The thrombolite is usually terminated by an erosional surface, which, however, may even cut down into the bryozoan/coralline algal-framestone. Therefore, the outermost ‘crust’ is frequently missing.

The zooecia of the bryozoa within the buildups are frequently inhabited by filamentous, dichotomously branching structures (diameter: 10–15 µm). These are most abundant in the peripheral zooecia and decrease inwards in abundance. The filaments are overgrown by a first cement generation. (Fig. 9b, c). Some of the bryozoan colonies show clear growth interruptions, marked by micritic bands. In these cases, the bifurcating structures are also very abundant from these levels inwards (Fig. 8c).

Within the framestone benthic biota are made up of gastropods, bivalves, foraminifera (mainly miliolids and very rare and small *Sinzowella*) and ostracods.

Voids, in particular those within bryozoan zooecia, are frequently rimmed by a first isopachous fibrous cement generation.

The terminal erosional surface of the buildups is covered by the oolite described above (Fig. 5b). Also the space between the frame within the buildups is frequently filled with oolites, proofing the still open framework of the buildups during oolite deposition.

## Discussion

### General background

The major 3rd-order sea-level drop at the Badenian/Sarmatian boundary (Ser3), the climatic deterioration due to the Middle Miocene Climate Transition (MMCT) and strong restriction of the open ocean connections of the Central Paratethys caused changes in biotic composition and a severe impoverishment of the marine fauna. The geodynamically induced reconnection with the Eastern Paratethys allowed the establishment of a rather uniform highly endemic Sarmatian mollusc and polychaete fauna and stenohaline organisms such as planktic foraminifera, radiolaria, corals and echinoids disappeared (e.g., Rögl 1998). This biotic reorganization is known as the Badenian–Sarmatian-extinction event (BSEE) (Harzhauser and Piller 2007).

During the early Sarmatian, water depth was overall low (30–50 m, 100 m maximum) pointing at inner neritic settings based on foraminiferal data for the Vienna Basin (Kranner et al. 2021a). Compared to the late Badenian, a slight shift from suboxic to more oxic epifaunal foraminiferal indicators has been observed and a mean salinity value of 35 psu varying from 31 to 40 psu and even more in coastal settings. Bottom water temperature shows a warm mean of 19 °C ranging from 14 to 25 °C what may be related to the overall shallowing of the basin (Kranner et al. 2021b).

### Reconstructing the sequence

**Fig. 10:**
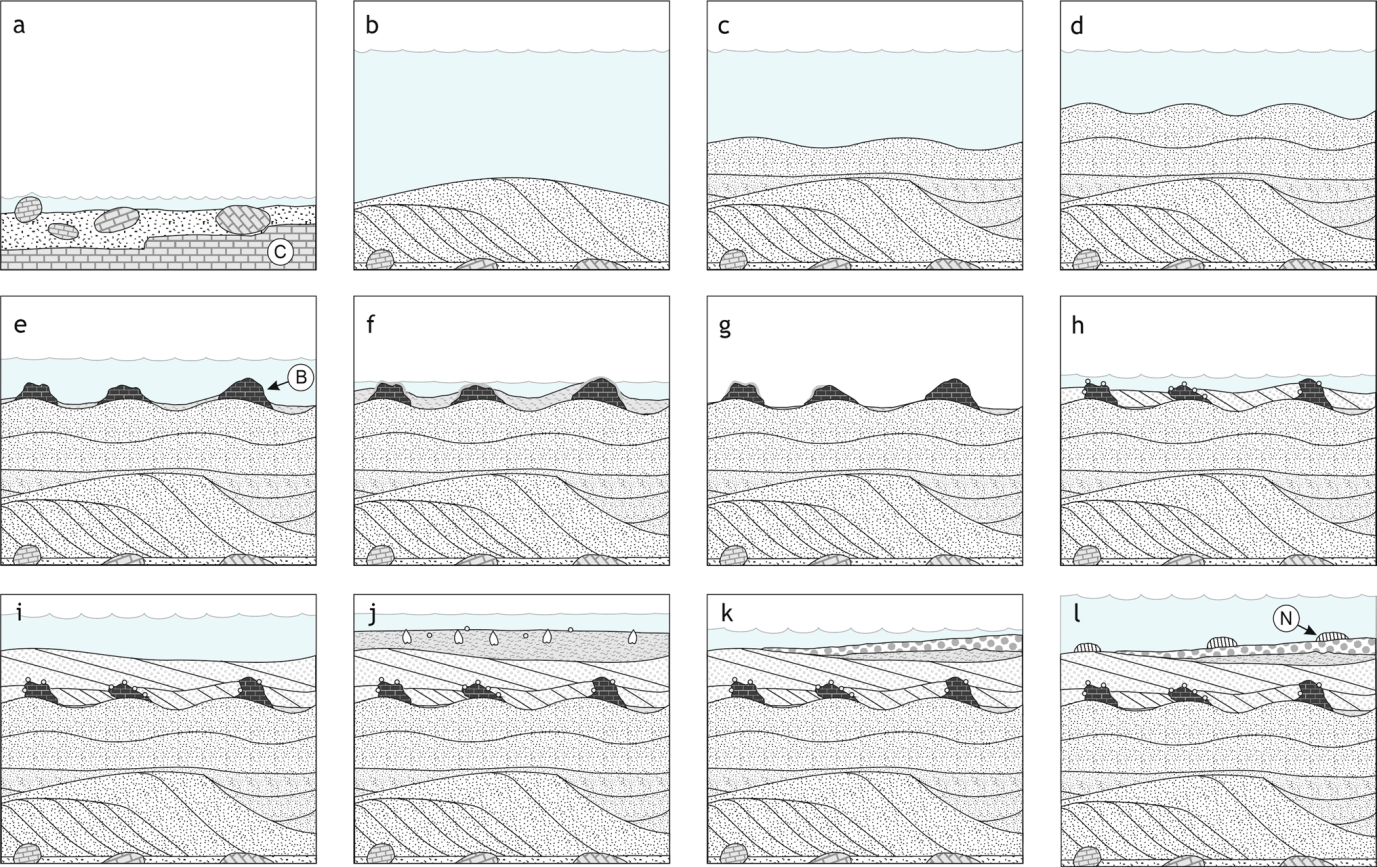
**a**–**f** Simplified Sarmatian evolution of the shoal in the Maustrenk area. The bryoherms developed during the terminal phase of the early Sarmatian transgression (*Mohrensternia* Zone) (C in **a**: Badenian coralline algal limestone, B in **e**: bryoherm). **g**–**j** Upper Sarmatian oolites of the *Ervilia* Zone covered the buildups after an erosional phase. **k**, **l** The last marine phase in the region is reflected by nubeculariid foraminifera *Sinzowella* buildups (N in l: *Sinzowella* buildup) (*Sarmatimactra* Zone)

The change from the Badenian corallinacean limestone to the lower Sarmatian carbonate sandstone is interpreted as emersion horizon (Fig. 10a). The subaerial exposure is ascribed to the 3rd-order sea-level lowstand at the Badenian/Sarmatian boundary (Harzhauser and Piller 2004a, b; Piller and Harzhauser 2005). The subsequent transgression in the earliest Sarmatian eroded and reworked these limestones producing coarse, well-rounded, detrital carbonate sands. These are arranged in large, cross-bedded sets representing subtidal sand dunes oriented in SSW–NNE direction (Fig. 10b, c). These bodies of sand dunes are overlain by sand sheets with large ripples (height: 20 cm, amplitude: 90 cm) exhibiting roughly the same orientation (Fig. 10d). On top of many ripples, individual carbonate buildups have settled (Fig. 10e, f). The depicted sedimentary sequence represents a deepening of the environment after flooding of the emersion horizon during the early Sarmatian. This forced the formation of sand dunes reflecting high hydrodynamic conditions. Upsection the magnitude of sand dunes decreases, and they are finally overlain by sand with more symmetrical wave-ripples reflecting already very shallow water. The rippled sand must have been stable and firm enough to be settled by the buildups. The abundant truncation and erosional features on top of the buildups can be related to another phase of subaerial exposure (Fig. 10g), described between the early and late Sarmatian removing most of the sediments of the *Elphidium hauerinum* Zone (Harzhauser and Piller 2004b) (Fig. 1). Subsequently, the space between the buildups was filled by cross-bedded oolite sand and the overlying bed covering the buildups is also a cross-bedded sand (Fig. 10h, i) made up predominantly of multi-layered, radial ooids (Fig. 5b). These oolitic sediments are of late Sarmatian age and correspond to the molluscan *Ervilia* Zone (Fig. 1). The latest stage of this phase is represented by clay with cardiids suggesting a muddy sluggish lagoon (Fig. 10j). After a minor erosional phase, a last transgression during the latest Sarmatian *Sarmatimactra* Zone (Fig. 1) established shallow lagoonal conditions with *Sinzowella* buildups in the Maustrenk area (Fig. 10k, l).

### The buildups

As pointed out above, the somewhat stabilized rippled sand has been settled by the crustose bryozoan *Cryptosula* and *Hydroides* serpulid worm tubes. These acted as a pioneer community which was succeeded by the massive bryozoan colonies of *Schizoporella.* These mostly nodular colonies formed a rigid skeletal frame frequently overgrown by crustose coralline algae. These coralline algae, although only filamentous or single-layered sheets, are interlayered with a dusty micrite which is interpreted to be of microbial origin. The coralline algae/microbial association produced columnar structures. These are finally overgrown by a thrombolite. The frame of this is made up of calcareous filaments which may be interpreted either as cyanobacteria or as eukaryotic algae embedded in clotted micrite. The cyanobacterial assignment would match modern as well as fossil examples (e.g., Pentecost and Riding 1996; Riding et al. 1991). Modern *Schizothrix* filaments and mats as being reported to be the main constituents of the modern Bahamian stromatolites are, however, not calcified (e.g., Reid et al. 1995; Feldmann and McKenzie 1998). In fact, the Sarmatian filaments fit much better those structures assigned to the green alga *Ostreobium* (e.g., Kobluk and Risk 1977) which is considered to be the most important frame-builder in the Bahamian thrombolites by Feldmann and McKenzie (1998).

This depicted general ecological succession clearly reflects a change in environmental conditions and since the major biota involved (bryozoans, serpulid worms) are filter feeders increasing nutrification is highly probable, the reduction in oxygen is reflected by microbial carbonate:

- At the very base of the buildups, basically, the *Cryptosula/Hydroides* pioneer community reflects colonization of the sediment surface by highly opportunistic organisms. Since this biota represents fouling organisms (see below) they are classified as opportunists (Callow and Callow 2011). The community is frequently overgrown by a first coralline algal crust, followed or accompanied by microbial carbonate. This succession may point to a slightly increasing nutrification and/or oxygen depletion.
- The next step with the growth of large *Schizoporella* colonies first proofs stable substrate on which these taxa may thrive. Again, an overgrowth by crustose coralline algae occurs, in these cases frequently in columnar growth form. Again, an increase in nutrients and a change in water chemistry might be indicated by this succession.
- The overlying “thicket” of eukaryotic (?) filaments and related clotted and pelleted sediments (thrombolite) implies a further increase in nutrient supply as does the final community of *Cryptosula* and spirorbid serpulids.
- The growth is terminated by erosion eventually during subaerial exposure of the buildups. The next flooding episode was coupled with a renewal of the water during transgression and allowed the production of thick oolite bars which are a widespread general feature of this transgression at the beginning of the late Sarmatian (Harzhauser and Piller 2004b; Piller and Harzhauser 2005). These migrating sand bars additionally truncated and asymmetrically eroded the buildups.

Looking for modern counterparts of bryozoan buildups, several examples can be cited. One is reported, but not described in great detail, from the Bahamas (Cuffey et al. 1977) which consists of two units: a lower bryozoan-rich and an upper coral-dominated part. The lower part seems to by widely comparable with the Sarmatian bioherms (except the distinctly lower diversity in the Sarmatian samples), the upper one clearly differs by the occurrence of corals. These buildups occur within a shifting oolite sand exposed to a high-energy level. The origin of the buildups is interpreted to be either caused by the topographical high built by the bryozoan-rich part which raises the buildup above the shifting oolite sand or by an environmental change from normal marine to slightly restricted waters, due to oolite shoal formation and movement (Cuffey et al. 1977; Cuffey and Fonda 1979). Although oolite sands are also present in our Sarmatian example these oolites are not contemporaneous with buildup formation but belong to a younger sedimentation cycle postdating a 4th-order sequence boundary (Harzhauser and Piller 2004b; Piller and Harzhauser 2005).

Another example of sub-recent/modern bryozoan–serpulid buildups is described from the Coorong lagoon in S Australia, first by Bone and Wass (1990) and later by Palinska et al. (1999), Scholz (2000) and Scholz et al. (2000, 2005). These are primarily composed by the laminated bryozoan genus *Conopeum* which is postmortally stabilized by cyanobacterial mats at the surface and fungal mats inside the zooecia. This alternation of bryozoa with microbial mats produces a biological lamination allowing to call these structures bryostromatolites. The controlling factors for these alternating biotas are considered to be seasonal fluctuations in salinity and water level. These bryostromatolites flourish in a highly restricted lagoon in an arid environment of hot, dry summers and cool, wet winters with a highly positive evaporation regime. Salinity ranges from normal marine in winter to 60 psu in summer, water temperature from 10 to 40 °C; when subaerially exposed sediment surfaces can reach up to 50 °C. Nevertheless, opportunistic *Conopeum* is able to form buildups in which it is intergrown with serpulid tubes (*Ficopomatus*). These bryozoans and serpulids are overgrown by microbial/fungal mats. Since bryozoans and microbial mats are not alive at the same time this structure is called a low-frequency type of a bryostromatolite (Palinska et al. 1999). The general mode of buildup construction is, therefore, similar to our Sarmatian examples. The main difference exists in bryozoan growth strategy: due to their ability of self-overgrowing (e.g., Scholz 2000; Scholz et al. 2000; Kaselowsky et al. 2005). *Conopeum* produces sheet-like lamina which are interlayered with microbial mats producing a finely laminated appearance of the resulting buildup, justifying or even requiring the term bryostromatolite (Scholz 2000; Scholz et al. 2000; Palinska et al. 1999). In our Sarmatian example, the volumetrically most important bryozoan is *Schizoporella* which exhibits nodular zoaria with irregular internal growth lamination. These growth laminae are not single sheets but include several layers of zooecia. The nodules are marginally covered and the zooecia are invaded by filaments best interpreted as fungal hyphae. Decreasing frequency of fungal hyphae from outer zoarial surfaces to internal zooecia proves that the fungi grew on already dead bryozoan surfaces and did not infect living colonies as described from some modern examples (Sterflinger et al. 2001). Also, this is a feature resembling Coorong bryostromatolites. The bryozoan nodules, attacked by fungi, are then overgrown by filamentous coralline algae intergrown with microbial mats. These red algal/microbial crusts display a columnar growth form and are subsequently overgrown by a thrombolite built by microbial mats as well as filamentous algae (?) altogether producing a clotted texture. Interlayered and on top of these mats sheet-like bryozoan *Cryptosula* as well as serpulid and spirorbid tubes are abundant which are, however, frequently eroded.

At the Mediterranean coast of France and in Sardinia microbialite buildups with bryozoan and worm tubes are reported from water depth of 0.5–1 m in lagoonal settings or in ponds (Saint Martin and Saint Martin 2015a, b). These buildups are exposed to large fluctuations in temperature and salinity and can even become aerially exposed or completely desiccated. The microbialite is dominated by the filamentous cyanobacteria *Scytonema*, bryozoans are represented by *Conopeum seurati*, serpulids by *Ficopomatus* and rare *Serpula* tubes; balanids are also frequently involved. Except of the cyanobacteria all other biota does not contribute to the frame of the buildups but are only accessory over- or intergrown in the cyanobacterial structures. From shallow subtidal environments (0.3–8 m) *Schizoporella errata* buildups are also described from the Ligurian Sea in the Mediterranean Sea. The buildups reach a width of 40 cm and a height of 20 cm (Cocito et al. 2000). Epibionts are represented by algae, hydroids, serpulids and barnacles. The growth morphology of *Schizoporella* ranges from encrusting to branching and is interpreted to be controlled by hydrodynamics.

Bryostromatolites from Netherlands’ costal lagoons/ponds, first described by Bijma and Boekschoten (1985), are made up by the monospecific bryozoan *Einhornia crustulenta* and cyanobacteria reaching a height of 1 m and isolated bryostromatolites may laterally coalesce and form linear reef structures of tens of meters. The bryostromatolites show a very high porosity and the cyanobacteria form a thrombolitic texture (Harrison et al. 2021). Salinity of the pond water is brackish with tidal fluctuations. The water contains toxic amounts of metals (arsenic, titanium) and periodically high sulfate concentrations indicating euxinic conditions. Cyanobacteria are considered to have formed during anoxic phases and overgrown bryozoans during more favorable conditions (Harrison et al. 2021, p 97).

The ecological succession within the Sarmatian buildups mirrors several cycles:

- One cycle can be observed on cm-scale in which *Schizoporella* nodules form the base which are overgrown/invaded by fungi and/or their hyphae (Sterflinger et al. 2001), followed by red algal/microbial mats and finally thrombolitic structures. This sequence occurs repeatedly within one buildup and may reflect short-time environmental changes, e.g., seasonal, annual or decennial fluctuations in temperature and/or salinity, as well as fluctuations in water depth but in particular a decrease in oxygenation as reported from modern bryostromatolites (Harrison et al. 2021).
- A second cycle is represented within the entire buildup. The succession starts with the *Cryptosula*/*Hydroides* pioneer community, followed by a level dominated by the *Schizoporella* nodules which is overlain by red algal/microbial mats and thrombolites. This sequence reflects a long-term succession which may be caused by an increase in nutrients and by a general change in water parameters. These may include changes in oxygen content creating anoxia, carbonate saturation and alkalinity besides those of temperature and salinity (Harrison et al. 2021). Nutrient and alkalinity increase are also reported for microbial carbonates covering coral successions in tropical reefs (e.g., Camoin et al. 1999, 2006; Cabioch et al. 2006).
- Due to the overall composition of the bioherms they should be descriptively called bryozoan–serpulid–algal–thrombolite buildups or simply bryozoan bioherms/bryoherms. Although good matches with modern (e.g., Palinska et al. 1999; Scholz et al. 2000, 2005; Harrison et al. 2021) and ancient bryostromatolites (e.g., Claussen et al. 2022) exist the amount of bryozoan framework and other biota is high. Also all mentioned major constituents—serpulids, algae, microbial structures—vary laterally considerably what is also not covered by the term bryostromatolite.

### Ecological requirements of modern *Hydroides*, *Cryptosula* and *Schizoporella*

The macroscopically most important constituents of the Sarmatian bryoherms are the polychaete *Hydroides* and the bryozoans *Cryptosula* and *Schizoporella*. All three genera are still widespread worldwide in warm coastal waters and are fouling organisms in harbors and on ships hulls (Pettengill et al. 2007; Abdelsalam and Ramadan 2008; Nedved and Hadfield 2009; Liu et al. 2017). These genera are tolerant against heavy eutrophication and pollution (Koçak and Kucuksezgin 2000; Koçak 2008; Zabin et al. 2010; Sandonnini et al. 2021). Especially, *Schizoporella* is considered as an ‘ecosystem engineer’ due to its ability to develop thick encrustations, which modify the original substratum (Loxton et al. 2017). For example, intertidal mudflats of the San Francisco Bay (California) became recently settled by *Schizoporella*, which developed spherical bryoliths of up to 20 cm diameter and buildups of 1 m lengths (Zabin et al. 2010). A change from oligiotrophic to strongly eutrophic conditions led to the formation of massive aggregates of *Hydroides/Serpula* in the Mar Menor lagoon in SE Spain (Sandonnini et al. 2021).

All three genera are also known for their wide salinity tolerance and some *Schizoporella* species dwell under mesohaline salinities as low as 15 psu (Powell 1970; Loxton et al. 2017). For the Sarmatian buildups, however, the frequent occurrence of *Hydroides* might indicate polyhaline waters because metamorphosis and the capability of successful settlement by *Hydroides* larvae decrease drastically at salinities below 22–26 psu (Qiu and Qian 1997; Pechenik et al. 2007; Liu et al. 2020). As euryhaline genera, all three genera, however, may also tolerate elevated salinities (e.g., *Hydroides* is reported to stand salinities up to 47 psu by Sandonnini et al. 2021). Serpulid bioherms with *Hydroides* were also documented from a Badenian hypersaline lagoon in the Austrian Oberpullendorf Basin shortly before stromatolites established in the lagoon (Harzhauser et al. 2014).

Based on the ecological requirements as filter feeders of the recent species of *Hydroides, Cryptosula* and *Schizoprella*, we assume that high nutrient load in a very shallow lagoon with polyhaline water has stimulated the formation of the buildups.

The descriptions of such or similar lower Sarmatian bryostromatolites in the literature, suggest that these buildups were common and widespread features in coastal waters of the Central Paratethys Sea at that time. Occurrences have been reported from the southern Vienna Basin, the Eisenstadt–Sopron Basin, the Styrian Basin and the Pannonian Basin (Harzhauser and Piller 2004a, b; Cornée et al. 2009; Hyzny et al. 2012). Consequently, the peculiar environmental conditions, which triggered the formation of bryoherms, characterized large parts of the Central Paratethys Sea during the early Sarmatian. Thus, we hypothesize that the early Sarmatian coastal ecosystems reflect a phase of highly variable salinity conditions and considerable eutrophication.

## Conclusion

Bryozoan-dominated bioherms of significant size (up to 50 cm in height) are described from lower Sarmatian (upper Middle Miocene) deposits of Lower Austria. They exhibit a clear internal ecological succession with (1) a *Cryptosula/Hydroides* pioneer community, (2) a *Schizoporella*-dominated community associated with coralline algae/microbial mats in columnar growth form which constitute the core of the bioherms, and (3) a termination community represented by thrombolites with calcareous filaments associated with *Cryptosula*-sheets and spirorbid tubes. This ecological succession is interpreted to reflect a general increase in nutrients and reduction of water circulation possibly creating anoxia. Internal successions of cm-scale are interpreted to reflect short-time fluctuations of environmental parameters. After an erosional phase, a renewed water body entered the area of deposition in the late Sarmatian and the buildups became covered and eroded by high-energy cross-bedded oolites.

The reasons for the origin of this type of buildups are manifold. One is the specific paleoceanographic and paleogeographic configuration during the late Middle Miocene of the Central Paratethys. After a distinct 3rd-order sea-level fall, the Central Paratethys became isolated from the Mediterranean and due to a change in circulation pattern and water chemistry stenohaline organisms disappeared. Despite the following sea-level rise, a re-immigration of stenohaline reef-building organisms, as for example corals, was prohibited and bryozoan were able to dominate bioherm formation (bryoherms) in alternation with microbial, algal and fungal communities in dependence from changing water parameters. The widespread occurrence of similar bryoherms in other Central Paratethyan basins suggests considerable eutrophication of coastal waters during the early Sarmatian.

## Data Availability

The materials used for this study are housed in the collection of the Geological- Paleontological Department of the Natural History Museum Vienna.
